# Concurrent Chemoradiotherapy-Driven Cell Plasticity by miR-200 Family Implicates the Therapeutic Response of Esophageal Squamous Cell Carcinoma

**DOI:** 10.3390/ijms23084367

**Published:** 2022-04-14

**Authors:** Yu-Cheng Lee, Cheng-Han Lin, Wei-Lun Chang, Wen-Der Lin, Jhih-Kai Pan, Wei-Jan Wang, Bor-Chyuan Su, Hsien-Hui Chung, Chen-Hsun Tsai, Forn-Chia Lin, Wen-Ching Wang, Pei-Jung Lu

**Affiliations:** 1 Graduate Institute of Medical Sciences, College of Medicine, Taipei Medical University, Taipei 110, Taiwan; yclee0212@tmu.edu.tw; 2 Institute of Clinical Medicine, College of Medicine, National Cheng Kung University, No. 35 Xiaodong Rd., Tainan 704, Taiwan; chanehan@gmail.com (C.-H.L.); weilun1@mail.ncku.edu.tw (W.-L.C.); lactose-2000@yahoo.com.tw (W.-D.L.); k610355@yahoo.com.tw (J.-K.P.); solonton650726@gmail.com (C.-H.T.); 3 School of Medicine, College of Medicine, I-Shou University, Kaohsiung 840, Taiwan; 4 Department of Internal Medicine, National Cheng Kung University Hospital, Tainan 704, Taiwan; 5 Department of Biological Science and Technology, Research Center for Cancer Biology, China Medical University, Taichung 404, Taiwan; cvcsky@cmu.edu.tw; 6 Department of Anatomy and Cell Biology, School of Medicine, College of Medicine, Taipei Medical University, Taipei 110, Taiwan; subc8265@tmu.edu.tw; 7 Preventive Medicine Program, Center for General Education, Chung Yuan Christian University, Taoyuan City 320, Taiwan; hsienhuichung@yahoo.com.tw; 8 Department of Pharmacy and Master Program, College of Pharmacy and Health Care, Tajen University, Pingtung 907, Taiwan; 9 Department of Radiation Oncology, College of Medicine, National Cheng Kung University Hospital, National Cheng Kung University, Tainan 704, Taiwan; fornchia@ncku.edu.tw; 10 Department of Surgery, Chi Mei Medical Center, No. 901, Zhonghua Rd., Yongkang Dist., Tainan 710, Taiwan; 11 Department of Clinical Medicine Research, National Cheng Kung University Hospital, Tainan 704, Taiwan

**Keywords:** acquired concurrent chemoradiotherapy resistance, microRNA, epithelial to mesenchymal conversion, esophageal cancer, cell plasticity

## Abstract

Esophageal squamous cell carcinoma (ESCC) is a common and fatal malignancy with an increasing incidence worldwide. Over the past decade, concurrent chemoradiotherapy (CCRT) with or without surgery is an emerging therapeutic approach for locally advanced ESCC. Unfortunately, many patients exhibit poor response or develop acquired resistance to CCRT. Once resistance occurs, the overall survival rate drops down rapidly and without proper further treatment options, poses a critical clinical challenge for ESCC therapy. Here, we utilized lab-created CCRT-resistant cells as a preclinical study model to investigate the association of chemoradioresistantresistance with miRNA-mediated cell plasticity alteration, and to determine whether reversing EMT status can re-sensitize refractory cancer cells to CCRT response. During the CCRT treatment course, refractory cancer cells adopted the conversion of epithelial to mesenchymal phenotype; additionally, miR-200 family members were found significantly down-regulated in CCRT resistance cells by miRNA microarray screening. Down-regulated miR-200 family in CCRT resistance cells suppressed E-cadherin expression through snail and slug, and accompany with an increase in N-cadherin. Rescuing expressions of miR-200 family members in CCRT resistance cells, particularly in miR-200b and miR-200c, could convert cells to epithelial phenotype by increasing E-cadherin expression and sensitize cells to CCRT treatment. Conversely, the suppression of miR-200b and miR-200c in ESCC cells attenuated E-cadherin, and that converted cells to mesenchymal type by elevating N-cadherin expression, and impaired cell sensitivity to CCRT treatment. Moreover, the results of ESCC specimens staining established the clinical relevance that higher N-cadherin expression levels associate with the poor CCRT response outcome in ESCC patients. Conclusively, miR-200b and miR-200c can modulate the conversion of epithelial–mesenchymal phenotype in ESCC, and thereby altering the response of cells to CCRT treatment. Targeting epithelial–mesenchymal conversion in acquired CCRT resistance may be a potential therapeutic option for ESCC patients.

## 1. Introduction

Cancer is a major public health problem and is the second leading cause of death worldwide. Esophageal cancer is a common cancer with approximately 572,000 cases; it is the seventh most common cause of cancer-related death, with an estimated 508,000 deaths in 2018 [1,2]. The prognosis in patients with esophageal cancer remains poor, the five-year survival is around 17–23% [3]. Histologically, esophageal squamous cell carcinoma (ESCC) is the major subtype of esophageal cancer and characterizes with extensive local growth and frequent metastases. Clinically, surgery is a common and first option for early-stage esophageal cancer treatment [4]. However, with the development of tumor progresses to advanced ESCC, for patients who have medically inoperable or unresectable tumors, radiation therapy exhibits the benefit to eliminate tumor size; in addition, the application of chemotherapy drugs such as cisplatin (DDP), 5-fluorouracil (5-FU), and doxorubicin (Dox) are the most widely available regimes to suppress tumor proliferation, local invasion, and metastasis in ESCC patients [5]. According to the treatment guideline in 2018, National Comprehensive Cancer Network (NCCN) recommends chemotherapy combined with radiation is the best option for esophageal cancer treatment. Concurrent chemoradiotherapy (CCRT) with or without surgery is a neoadjuvant treatment for locally advanced ESCC in the last decade [6,7]. However, not all patients benefit from or show a good response to CCRT; accumulating evidence exists that acquired resistance to CCRT occurs during the interval of treatment cycle and that leads to treatment failure and cancer relapse, which is called concurrent chemoradiotherapy resistance (CCRT^R^) [8,9]. Accordingly, it is crucial to elucidate the potential mechanisms of CCRT resistance and develop novel therapeutic strategy in ESCC.

miRNAs belong to small non-coding RNAs, which can directly bind to specific mRNAs to suppress the expression of their corresponding target proteins via promoting mRNAs degradation or repressing their translation. miRNAs play critical roles to maintain numerous normal physiology events, e.g., cell proliferation, differentiation, development and immune response [10,11]. To adapt environmental stimuli, the biosynthesis and turnover of miRNAs are dynamic, but have to be tightly controlled. Aberrant miRNAs expressions develop multiple pathological diseases, including neurodegenerative diseases, tumorigenesis, cancer metastasis and drug resistance in cancer therapy [11,12,13,14]. From the aspects of disease treatment, the concept of targeting miRNAs is an emerging therapeutic strategy and currently employed in preclinical and clinical trials [15,16]. Moreover, miRNAs expression level also contribute to treatment responses and as predictive biomarkers for therapeutic efficacy [17,18].

Cell plasticity refers to the potential of one type of cells develop into a different type of cells in response to microenvironmental alterations and aberrant stimulation [19]. The regulations of cell morphological change between epithelium and mesenchyme are complicated and mainly involve in normal developmental biology, such as tissue regeneration and wound repair [20]. Several master transcription factors, e.g., snail and slug, have been shown to regulate the conversion of epithelium to mesenchyme [21,22]. EMT features with hallmarks of downregulated epithelial marker, E-cadherin and upregulated mesenchymal markers, N-cadherin and vimentin [23,24]. Snail and slug can downregulate E-cadherin expression by repressing the E-cadherin promoter activity. Growth factors (e.g., TGF-β) and environmental stress (e.g., hypoxia) can trigger epithelial to mesenchymal phenotypes [25,26], indicating cell plasticity is regulable and reversible in responding to extrinsic stimulation. Increasing evidences revealed when cells re-adopt mesenchymal characteristic, it could play several pathological roles in developing human diseases, including fibrosis, tumor formation, metastasis, and drug resistance. These phenomena suggested the intervention of epithelial–mesenchymal conversion may potentially block pathological progression [27,28,29,30].

ESCC patients harbor poor response to CCRT or acquire CCRT resistance during the therapeutic course remains a clinical challenge. The knowledge of chemoradioresistance and miRNA-mediated EMT, particular in advanced ESCC, is lacking. Uncovering the potential mechanisms of miRNA-mediated EMT and chemoradioresistance in ESCC will be important both in biology and in therapeutic promises. In this study, we established a pre-clinical CCRT resistance model to mimic the clinical problem regarding ESCC patients acquire chemoradioresistance during the treatment course. To date, accumulating evidences revealed the deregulation of miRNAs involves in pathological events, our results revealed the dynamic change of cell morphology from epithelial to mesenchymal phenotype during the development of CCRT resistance. Such cell plasticity alteration was mediated by the down-regulation of miR-200 family members. Moreover, manipulating miR-200 family members expressions in non-CCRT resistance ESCC cells, it can modulate the switch of epithelial to mesenchymal phenotype and impair cells viability to CCRT response. Collectively, these results implied the cause-effect of miRNA-mediated EMT and chemoradioresistance. Moreover, targeting miRNA as a therapeutic approach is under phase I/II clinical trial, our findings may provide the proof that targeting miRNA to modulate cell plasticity could as a therapeutic approach to improve CCRT response in ESCC.

## 2. Results

### 2.1. Acquired Concurrent Chemoradiotherapy Resistance (CCRT^R^) ESCC Cell Lines Establishment

**Figure 1:**
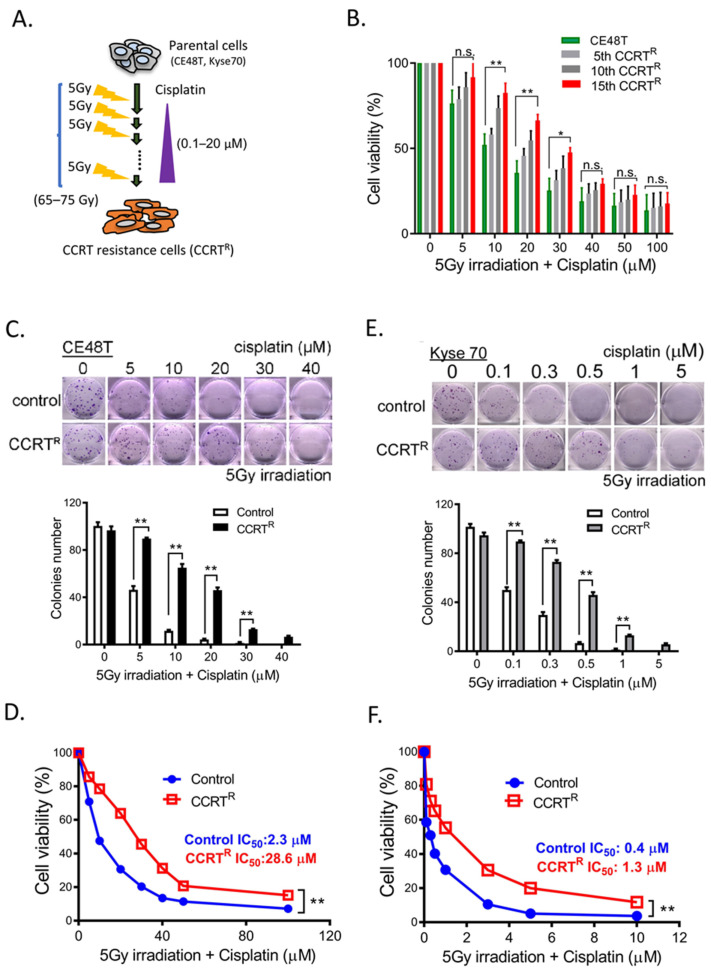
Establish acquired concurrent chemoradiotherapy resistance (CCRTR) ESCC cell lines and validate their viability to CCRT treatment. (**A**) A schematic diagram of two chemoradioresistant ESCC cell lines (CE48T and Kyse70) established by fractionated irradiation (5 Gy) and cisplatin treatment. Total effective dosage of irradiation exposure was 65–75 Gy. (**B**) Cell viability was examined in CCRT-selected CE48T cells. Different CCRT intensity-selected CE48T cells (5th, 10th and 15th) and control cells were treated with the indicated CCRT conditions, 5 Gy irradiation and various concentrations of cisplatin (0, 5, 10, 20, 30, 40, 50 and 100 μM) to determine their CCRT response by MTT assay. These three independent cell lines harbored a consistent resistance potential but differential resistance ability to CCRT treatment. (**C**) Validation of chemoradioresistant ability in acquired CE48T CCRT-resistance line (CCRTR) by colony formation assay. CE48T parental control and CCRTR cells were treated with the combination of 5 Gy irradiation exposure and indicated concentrations of cisplatin (0, 5, 10, 20, 30, 40 μM). At the end of 12 days incubation period, viable cell colonies were fixed, stained with crystal violet, and calculated. Upper panel: Representative images of viable colonies after CCRT treatment; lower panel: Quantification of CCRT-mediated colonies formation in control and CCRTR cells at 12 days post-CCRT treatment. (**D**) Examine the effect of CCRT treatment on cell viability using CE48T control and CCRTR cells by MTT assay. The 1 × 10^4^ control and CCRTR cells were plated into a 96-well plate, cells were treated with 5 Gy irradiation combined with the indicated concentrations of cisplatin (0, 5, 10, 20, 30, 40, 50, and 100 μM) for 48 h. Relative quantification of cell viability in control and CCRTR cells post 48h-CCRT treatment compared to cells without CCRT treatment were shown. The IC50 to cisplatin is 2.3 μM in control cells and 28.6 μM in CCRTR cells. (**E**) Validation of Chemoradioresistant ability in acquired Kyse70 CCRT-resistance line (CCRTR) by colony formation assay. Experimental protocol was similar to (C), whereas the treatment concentrations of cisplatin were 0, 0.1, 0.3, 0.5, 1, and 5 μM. Representative images of viable colonies at 14 days post-CCRT treatment (top) and quantification of CCRT-mediated colonies formation in control and CCRTR cells (bottom) were shown. (**F**) Examine the effect of CCRT treatment on cell viability using Kyse70 control and CCRTR cells by MTT assay. The 1 × 10^4^ control and CCRTR cells were plated into a 96-well plate, cells were treated with 5 Gy irradiation combined with the indicated concentrations of cisplatin (0, 0.1, 0.3, 0.5, 1, and 5 μM). The IC50 to cisplatin is 0.4 μM and 1.3 μM in control and CCRTR of Kyse70 cells, respectively. * *p* < 0.05, ** *p* < 0.01, n.s.: not significant.

Platinum-based regimen (e.g., cisplatin) is widely recommend for the first-line option of advanced ESCC treatment in current guidelines [31]. In order to investigate the possible underlying mechanisms of CCRT^R^, we first employed irradiation combined with the anti-cancer agent cisplatin to establish acquired concurrent chemoradiotherapy resistance (CCRT^R^) ESCC cell lines, including CT48T and Kyse70 cells. Figure 1A showed the schematic diagram of irradiation fractionation schemes and cisplatin treatment were used. Briefly, CE48T and Kyse70 cells were delivered a total dose of 65 to 75 Gy by multi-fraction regimen of 5 Gy per time and 0.1–20 μM cisplatin throughout the selection period. In-between of CCRT treatment, 5th, 10th, and 15th CCRT-selected CE48T cells were collected and subjected to MTT assay to examine the effect of irradiation and cisplatin treatment on cell viability. Figure 1B showed all three CCRT-selected cells exhibited higher cell viability than non-CCRT treatment control cells upon CCRT treatment. Moreover, the 15th CCRT-selected CE48T cells harbored stronger resistance capacity than the 5th and 10th CCRT-selected cells, suggesting the resistance capacity of CE48T cells to CCRT treatment was increased with the CCRT treatment cycle. At the end of CCRT selection cycle, to validate the established CCRT resistance cells harbored poor response to CCRT, CCRT-selected CE48T, Kyse70 cells and their corresponding parental control cells were treated with 5 Gy irradiation combining with various concentrations of cisplatin, and then cells were subjected to colony formation assay to examine their chemoradioresistant ability (Figure 1C,E). The result of Figure 1C revealed that cells exposed to 5 Gy irradiation and cisplatin (0, 5, 10, 20, 30, 40 μM) treatment, CE48T parental control cells exhibited a markedly impaired colony-forming capacity upon 5 Gy irradiation with 10 μM cisplatin treatment (~10 colonies formation), whereas CCRT-selected CE48T cells had remained ~70 colonies formation at the same treatment condition compared to control cells. Additionally, similar experiments were performed in Kyse70 parental control and CCRT-selected cells (Figure 1E), the discrepancy was the effective concentration of cisplatin in Kyse70 (0, 0.1, 0.3, 0.5, 1, 5 μM), since Kyse70 cells displayed a greater cisplatin sensitivity than CE48T cells. The result in Figure 1E showed CCRT-selected Kyse70 (~60 colonies) exhibited a higher colony-forming ability than control cells (~5 colonies) at the same 0.5 μM cisplatin treatment. Furthermore, to estimate the potency of cisplatin in CCRT-selected CE48T and Kyse70 cells, CCRT-selected CE48T and Kyse70 cells and their control cells were treated with the indicated concentrations of cisplatin and then subjected to MTT assay, respectively (Figure 1D,F). Figure 1D showed compared to control cells, CCRT-selected CE48T cells acquired approximately 12-fold increase in the half maximal inhibitory concentration (IC_50_) of cisplatin-induced cell death from 2.3 μM to 28.6 μM. Moreover, the IC_50_ of cisplatin-induced cell death in CCRT-selected Kyse70 cells was 1.3 μM, at least approximately 3-fold increase compared to Kyse70 control cells (IC_50_: 0.4 μM). Taken these results together, these biological results of colony formation assay and cell viability clearly indicated acquired concurrent chemoradiotherapy resistance (CCRT^R^) ESCC cell lines were successfully established in CE48T and Kyse70 cells.

### 2.2. Feature of Epithelial-Mesenchymal Conversion in CCRT^R^ ESCC Cells

**Figure 2:**
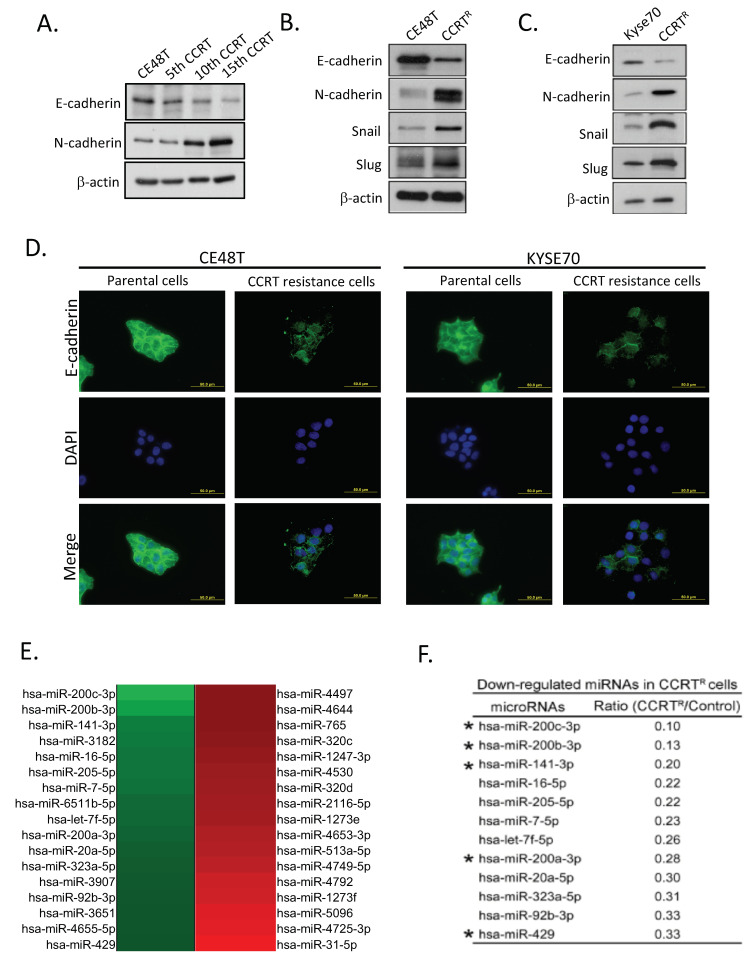

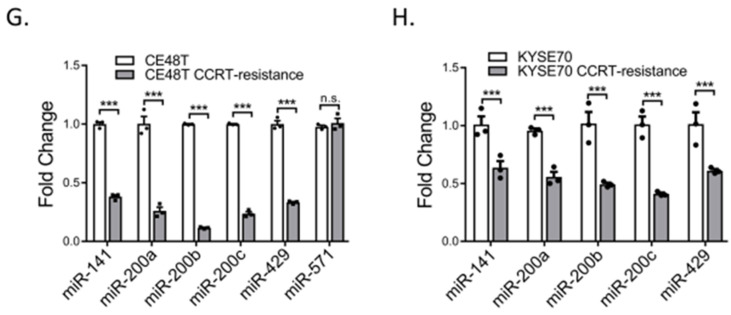
Acquired CCRT resistance cells exhibit the conversion of epithelium to mesenchyme and down-regulate miR-200 family expressions. (**A**) CCRT resistance ability correlated with the status of epithelial–mesenchymal conversion in three independent CE48T CCRT-resistance lines. Total cell lysates from CE48T control and three independent CCRT^R^ cells as showed in Figure 1B were collected and examined by Western blot analysis using anti-E-cadherin and N-cadherin antibodies. β-actin was used as loading control. (**B**) Examination of E-cadherin and mesenchymal-related markers (N-cadherin, snail, slug) expressions. Total cell lysates of CE48T control and CCRT^R^ cells were harvested and then subjected to Western blot analysis using the indicated antibodies. β-actin was used as loading control. (**C**) Total cell lysates of Kyse70 control and CCRT^R^ cells were examined by Western blot analysis using the indicated antibodies as described in (**B**). (**D**) Immunofluorescence analysis of E-cadherin expression in CE48T and Kyse70 parental and CCRT resistance cells. E-cadherin expression was shown in green color and DNA staining in blue. Scale bar: 50 µm. Compared to parental cells, the immunofluorescence intensity of E-cadherin expression was reduced and its subcellular location in the cell surface was impaired in CCRT resistant cells. (**E**) Identification of potential microRNAs by Human OneArray system (HOA 6.1). Total RNA from control and CE48T CCRT^R^ cells were extracted and then subjected to microRNA microarray to identify differential miRNAs expressions in control and CE48T CCRT^R^ cells. Red and green colors indicate high and low expression, respectively. (**F**) A list of downregulated miRNAs in CE48T CCRT^R^ cells. Ratio means miRNAs expression of CE48T CCRT^R^ cells normalized to control cells. The 12 selected miRNAs showed at least a 3-fold lower expression in CE48T CCRT^R^ cells. * indicated miRNAs belong to miR-200 family. (**G**,**H**) Validation of miRNA targets expression in CE48T and Kyse70 CCRT^R^ cells by qRT-PCR analysis, respectively. The relative expression levels of five miR-200 family members (miR-200a, miR-200b, miR-200c, miR-141, and miR-429) were examined in CE48T and Kyse70 CCRT^R^ cells, respectively, and normalized to control cells. Relative fold-change of each selected miRNA was shown, compared to control cells marker as 1. miR-571 was not differentially expressed in control and CCRT^R^ cells, and using as an experimental control. * *p* < 0.05, *** *p* < 0.001, n.s.: not significant.

It is interesting that during the interval of cells exposed to CCRT treatment, we observed that CCRT-treated cells progressively presented mesenchymal-like morphology in comparison with non-CCRT treatment control cells. Subsequently, we carefully confirmed this interesting observation and validated it by molecular levels. Total cell lysates of post 5th, 10th, and 15th CCRT treatment were collected and subjected to Western blot analysis. Specific antibodies against E-cadherin and N-cadherin were used to examine the status of epithelial–mesenchymal conversion. The results in Figure 2A strikingly showed the expression of epithelial marker-E-cadherin was lost accompanied with the increased frequency of CCRT treatment. Conversely, the mesenchymal marker-N-cadherin expression was up-regulated in CCRT-treated CE48T cells. Put the results of Figure 1B that differential cell viability post 5th, 10th, and 15th CCRT treatment together with this observation, it strongly implied the status of mesenchymal characteristic in cells might contribute to a poor response to CCRT treatment and can mirror the response to CCRT.

Subsequently, we examined whether the suppression of E-cadherin was mediated by the up-regulation of snail and slug in CCRT^R^ cells. Total cell lysates of CE48T parental control and CCRT^R^ cells were collected and subjected to Western blot analysis (Figure 2B). The results revealed compared to parental control cells, CE48T CCRT^R^ cells expressed lower E-cadherin, whereas the expressions of snail and slug were increased, as well as in N-cadherin. Similar results were obtained in Kyse70 CCRT^R^ cells (Figure 2C). Furthermore, the reduction of E-cadherin expression in CCRT^R^ cells was confirmed by immunofluorescence assay. Figure 2D revealed both CE48T and KYSE70 CCRT resistance cells, the intensity of E-cadherin staining (green color) was obviously decreased when compared to parental cells. Moreover, the location of E-cadherin in cell surface was reduced, indicating the intercellular junctions were impaired upon the loss of E-cadherin expression. Conclusively, these two independent CCRT^R^ lines established the association of EMT status and acquired concurrent chemoradiotherapy resistance by showing ESCC tumor cells with mesenchymal feature harbor poor response to CCRT treatment.

### 2.3. Down-Regulation of miR-200 Family Members in CCRT Resistance Cells

MicroRNAs (miRNAs) involve in many pathological events. To explore whether miRNAs play potential functions responding to CCRT resistance, NCode™ human miRNA microarray V3 was performed using total RNA extractions from CE48T control and CCRT^R^ cells. The heatmap in Figure 2E showed miRNAs expression profiles in control relative to CE48T CCRT^R^ cells. Green and red color represented down-regulated and up-regulated miRNAs, respectively. Analysis of these array date revealed a bunch of distinct miRNAs were down-regulation in CE48T CCRT^R^ cells relative to control cells. Figure 2F showed the list of potential miRNAs were down-regulated in CE48T CCRT^R^ cells, including miR-7, let-7f, miR-16-5p, miR-200a, miR-200b, miR-200c, miR-141, and miR-429, etc. miR-200 family members (miR-200b and miR-200c) were first selected and validated in our model based on the following criteria. First, miR-200b and miR-200c were the Top 2 candidates with the most down-regulated expression levels in CCRT^R^ cells. Second, miR-200 family consists of five members, in addition to miR-200b and miR-200c, the other three members such as miR-141, miR-200a, and miR-429 were also down-regulated in CCRT^R^ cells. Such consistent downregulation in all five members, suggesting miR-200 family could be important to modulate CCRT resistance. Third, the interesting phenotype of CCRT^R^ cells adopted mesenchymal feature in Figure 2A–D agreed with the biological role of miR-200 family regulates epithelial cell plasticity. Therefore, these increased the possibility that miR-200 family implicated in CCRT resistance, in particular miR-200b and miR-200c.

Subsequently, to further confirm miR-200 family members were significantly downregulated in CCRT^R^ cells. The validations of individual miR-200 family members expressions were examined in two independent CCRT^R^ cell lines, including miR-141, miR-200a, miR-200b, miR-200c, and miR-429. In CE48T CCRT^R^ cells, quantitative RT-PCR (qRT-PCR) results revealed the expression of these distinct miR-200 family members were indeed found to be decreased (Figure 2G). miR-200b and miR-200c were obviously reduced by ~75 to 80% in resistance cells compared to control cells. Similarly, the reduced expressions of distinct miR-200 family members were also observed in Kyse70 CCRT^R^ cells (Figure 2H). Collectively, these validated results in two independent ESCC CCRT^R^ cell lines firmly confirmed the miRNA microarray data and illustrated miR-200 family expressions were impaired in CCRT resistance cells.

### 2.4. miR-200 Family Members Can Modulate CCRT Response

**Figure 3:**
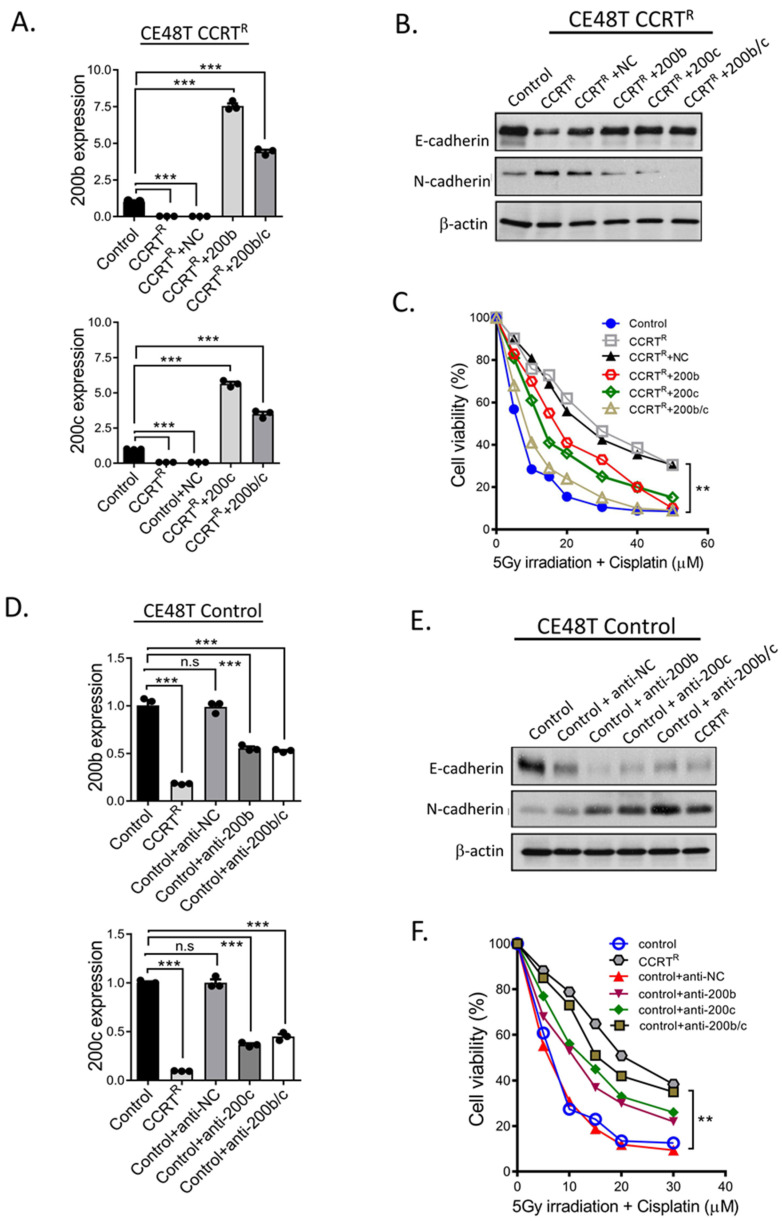
The role of miR-200b and miR-200c-mediated CCRT responses in CE48T cells. (**A**) Exogenous control miRNA, miR-200b, miR-200c, and miR-200b/c precursors were transfected into CE48T CCRT^R^ cells, respectively. To confirm transfection efficiency, miR-200b (top panel) and miR-200c (bottom panel) expressions were examined by qRT-PCR. (**B**) Examination of E-cadherin and N-cadherin expressions in CE48T CCRT^R^ cells with indicated miRNA precursors expression. After 48h transfection, total cell lysates from control, CCRT^R^ and control miRNA, miR-200b, miR-200c, and miR-200b/c-expressed CCRT^R^ cells were harvested and subjected to Western blot. (**C**) Exogenous miR-200b, miR-200c, and miR-200b/c overexpression sensitized CCRT^R^ cells to CCRT treatment. CE48T CCRT^R^ cells were transfected with indicated miRNA precursors as described in (**A**). After 48h transfection, CE48T CCRT^R^ with miR200b, 200c, and 200b/c overexpression were exposed to CCRT treatment with 5 Gy irradiation and indicated concentrations of cisplatin (0, 5, 10, 15, 20, 30, 40, 50 μM). Cell viability was examined by MTT assay. The relative percentage of viable cells were shown comparing to the cells without CCRT treatment as 100%. (**D**) The indicated anti-miRNA oligonucleotides (NC, 200b, 200c, and 200b/c) were transfected to CE48T control cells. After 48h transfection, miR-200b (top panel) and miR-200c (bottom panel) expressions were validated by qRT-PCR. Relative fold-change of 200b, 200c, and 200b/c expressions compared to control and anti-miRNA control groups were shown. (**E**) E-cadherin and N-cadherin expressions in total cell lysates from control, anti-NC, anti-200b, anti-200c, and anti-200b/c miRNA oligonucleotides-transfected CE48T control cells were examined by Western blot analysis. β-actin was used as loading control. (**F**) The effect of CE48T control cells expressed anti-miR200b, anti-200c, and anti-200b/c oligonucleotides to CCRT treatment. miR-200b, 200c, and 200b/c expression levels-impaired CE48T control cells were subjected to CCRT treatment as described in (**C**). After 48h incubation, MTT assay was used to examine cell viability. ** *p* < 0.01, *** *p* < 0.001, n.s.: not significant.

The results in Figure 2 revealed tumor cells with mesenchymal feature harbors poor response to CCRT treatment, and CCRT resistance cells displayed significantly reduction in miR-200 family members expressions. Collectively, it implied miR-200 family mediated mesenchymal feature might confer tumor cells with resistance capacity to CCRT, and to intervene miR-200 family expressions in cells could potentially modulate the sensitivity to CCRT response. To address this possibility, miR-200b and miR-200c expressions were manipulated in control and CE48T CCRT^R^ cells. E-cadherin and N-cadherin were used to represent cell epithelial or mesenchymal feature, MTT assay was used to determine the ability of cells respond to CCRT treatment. miR-200b, miR-200c, and miR-200b/c precursors were initially transfected into CE48T CCRT^R^ cells to restore their expressions, respectively, since CE48T CCRT^R^ cells expressed lower miR-200b and miR-200c than control cells (Figure 2G). qRT-PCR results in Figure 3A confirmed miR-200b and miR-200c were effectively re-expressed in CE48T CCRT^R^ cells. In addition, ectopic miR-200b, miR-200c, and miR-200b/c overexpression led to CE48T CCRT^R^ cells rescued E-cadherin expression and reversely decreased N-cadherin level (Figure 3B). This result suggested the conversion of mesenchymal to epithelial phenotype could control by miR-200 family. Next, to determine such conversion by miR-200 family could improve the poor response of CE48T CCRT^R^ cells to CCRT treatment. Accordingly, CE48T CCRT^R^ cells with miR-200b, miR-200c, or miR-200b/c overexpression were subjected to CCRT treatment, and examined the cell viability by MTT assay. Figure 3C revealed compared to CCRT^R^ cells or CCRT^R^ cells with anti-NC miRNA transfection as experimental control, the survival curve of CCRT^R^ cells with miR-200b and miR-200c overexpression shifted and approached control curve. Moreover, resistance cells exerted an additional inhibitory effect to CCRT treatment when miR-200b and miR-200c (miR-200b/c) co-overexpression in CE48T CCRT^R^ cells. These results suggested re-expression of miR-200b and miR-200c in CE48T CCRT^R^ cells could convert cells to epithelial feature, and this conversion effectively promoted the re-sensitization of resistance cells to CCRT treatment.

To further investigate the role of miR-200b and miR-200c-mediated CCRT response was through EMT modulation; for this purpose, CE48T control cells with higher E-cadherin were utilized and transfected with anti-miRNA oligonucleotides (NC, miR-200b, miR-200c, and miR-200b/c) to against endogenous miR-200b and miR-200c expressions. As shown in Figure 3D, miR-200b and miR-200c levels in transfected CE48T control cells were confirmed by qRT-PCR, the results validated endogenous miR-200b and miR-200c expressions were suppressed after transfection. In addition, CE48T control cells with repressed miR-200b and miR-200c expressions exhibited a reduction in E-cadherin expression and accompanied by an increase in N-cadherin (Figure 3E). Subsequently, these CE48T cells with impaired E-cadherin suppression by anti-miR-200b, miR-200c, and miR-200b/c oligonucleotides were treated with CCRT to examine their cell viability. Figure 3F clearly revealed these cells under CCRT treatment, their survival curve migrated to the direction of CCRT^R^ cells survival curve. Collectively, these results suggested the effect of anti-miR-200b and anti-miR-200c expression could modulate cells to harbor mesenchymal feature, thereby conferring CE48T control cells with resistant ability to CCRT treatment, which was similar to CCRT^R^ cells.

### 2.5. Additional Evidences Supporting miR-200 Family Manipulation Affects CCRT Response

**Figure 4:**
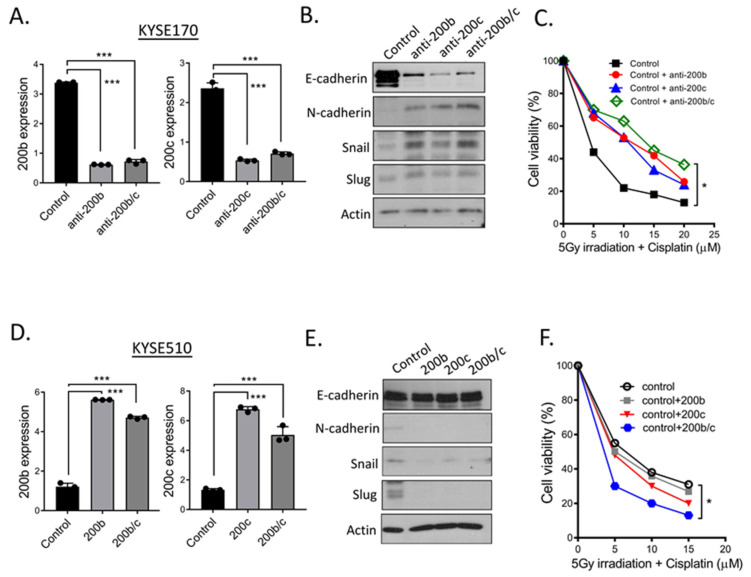
Manipulating miR-200b and miR-200c expressions in Kyse170 and Kyse510 cells alter cell epithelial–mesenchymal feature and modulate cell sensitivity to CCRT treatment. (**A**) miR-200b and miR-200c levels were manipulated by anti-miR-200b and miR-200c oligonucleotides transfection in Kyse170 cells, the reduction of miR-200b and miR-200c expressions were confirmed by qRT-PCR analysis. (**B**) Examination of E-cadherin, N-cadherin, snail, and slug expressions in anti-miR-200b, miR-200c, and miR-200b/c oligonucleotides-transfected Kyse170 cells. Total cell lysates were collected and subjected to Western blot using indicated antibodies (anti-E-cadherin, N-cadherin, snail, and slug). β-actin was determined as loading control. (**C**) miR-200b and miR-200c suppression modulate Kyse170 cells resisting to CCRT treatment. Control and anti-miR-200b, anti-miR-200c, or anti-miR-200b/c oligonucleotides-transfected Kyse170 cells were plated into a 96-well plate, treated with 5 Gy radiation and at the indicated concentrations of cisplatin (0, 5, 10, 20, 30, 40, 50, and 100 μM). After 48h incubation, cell viability was determined by MTT assay. Relative percentage of viable cells compared to cells without CCRT treatment were shown. (**D**) Kyse510 parental cells were transfected with control, miR-200b, miR-200c and miR-200b/c precursors, respectively. qRT-PCR analysis was used to confirm ectopic miR-200b, miR-200c and miR-200b/c expressions in Kyse510 parental cells. (**E**) Total cell lysates of transfected Kyse510 parental cells from (**D**) were collected and subjected to Western blot using indicated antibodies (anti-E-cadherin, N-cadherin, snail, and slug). β-actin was used as loading control. (**F**) Control and miR-200b, miR-200c or miR-200b/c precursors-transfected Kyse510 were subjected to MTT assay to examine the effect of CCRT treatment on their cell viability. Treatment conditions were described as in (**C**). Relative percentage of viable cells compared to cells without CCRT treatment were shown. * *p* < 0.05, *** *p* < 0.001.

Previously, we utilized CE48T cells as a model to investigate the pathological role of miR-200 family in CCRT resistance. To further characterize epithelial–mesenchymal feature could modulate the response to CCRT, and additionally, the morphological alteration by miR-200b and miR-200c was a broad mechanism to develop acquired resistance in ESCC, two ESCC cell lines-Kyse170 and Kyse510 were furtherly used in our study (Figure 4). The expression levels of miR-200b and miR-200c were manipulated by transfecting either miR-200b and miR-200c precursors or anti-miR-200b and anti-miR-200c oligonucleotides in cells. qRT-PCR results in Figure 4A validated the suppression capacity of miR-200b and miR-200c by anti-miR-200b and anti-miR-200c oligonucleotides in Kyse170 cells. Western blot analysis in Figure 4B confirmed the similar results in Figure 3E that suppressing miR-200b and miR-200c expressions, ESCC cells expressed higher snail and slug, and accompanied by the down-regulation of E-cadherin and up-regulation of N-cadherin. Additionally, such a mesenchymal alteration in cells could impair the sensitivity to CCRT treatment (Figure 4C).

On the other hand, miR-200b, miR-200c, and miR-200b/c precursors were transfected into Kyse510 parental control cells to reinforce miR-200b, miR-200c, and miR-200b/c levels in cells. qRT-PCR results and Western blot analysis separately revealed miR-200b and miR-200c levels were increased in Kyse510 parental control cells after transfection (Figure 4D); a reduction in N-cadherin expression, as well as in snail and slug, and an increased in E-cadherin expressions (Figure 4E). Besides, miR-200b, miR-200c, and miR-200b/c overexpression could modulate cells with epithelial feature and sensitize Kyse510 parental control cells to CCRT response (Figure 4F). These results were similar to the outcome of CE48T CCRT^R^ with miR-200b, miR-200c, or miR-200b/c overexpression (Figure 3A–C). Conclusively, these reciprocal experiments revealed miR-200 family mediated the alteration of epithelial–mesenchymal feature can affect cells respond to CCRT treatment. Moreover, it also implied the status of epithelial–mesenchymal could be used to predict CCRT response in ESCC patients.

### 2.6. N-cadherin Staining Levels Correlate with ESCC Patients Respond to CCRT

**Figure 5:**
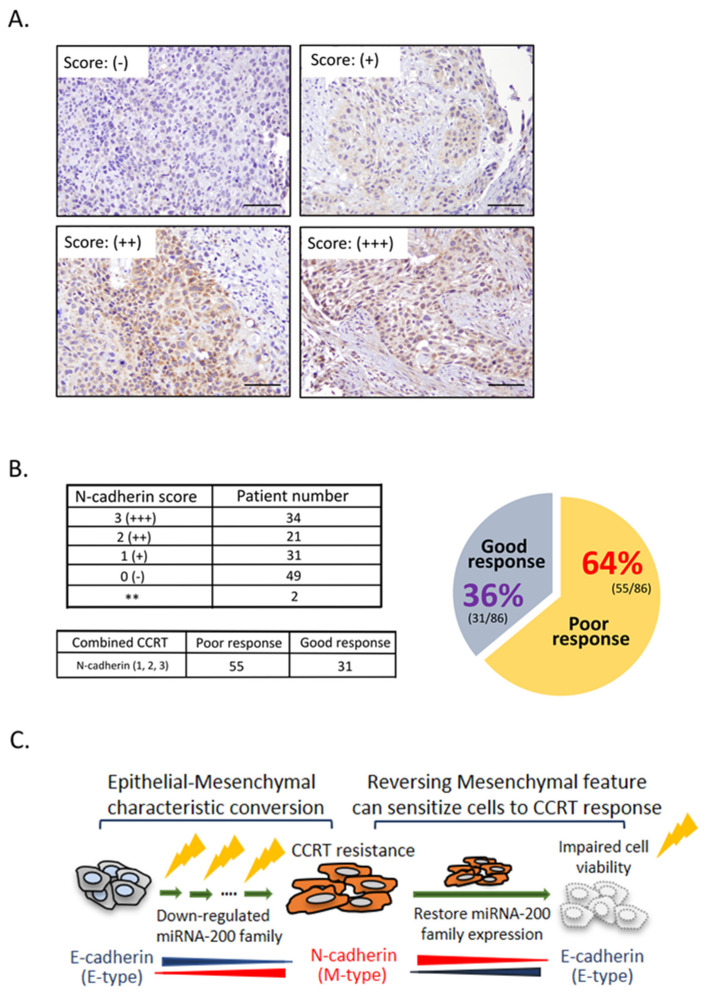
N-cadherin status is a potential marker to predict CCRT response in ESCC patients. (**A**) 137 endoscopic biopsy ESCC tissue samples before CCRT treatment were collected and subjected to immunohistochemistry analysis using anti-N-cadherin antibody. Representative photos showed the scoring criteria by staining intensity: 0, negative (-); 1+, positive; 2+, moderately positive; and 3+, strongly positive. Scale bar: 50 µm. (**B**) Higher N-cadherin-expressed ESCC tissue specimens exhibited a poor response to CCRT treatment. In total, 86 of 137 tissue samples expressed higher N-cadherin, 55 of 86 tissue samples (64%) were poor response to CCRT, whereas 31 of 86 tissue samples (36%) showed good response to CCRT treatment. ** mean tissue specimen was broke. (**C**) Schematic diagram illustrating the potential mechanism of ESCC acquire CCRT resistance. During the interval of CCRT treatment, down-regulated miR-200 family modulates the conversion of epithelial to mesenchymal feature that enables cells acquire resistance capacity to CCRT treatment. Rescue miR-200 family expression can convert CCRT resistance cells to epithelial feature and modulate the sensitivity to CCRT treatment.

Subsequently, in order to establish the clinical relevance of acquired CCRT resistance with the conversion of epithelial to mesenchymal feature; ideally, a paired pre- and post-CCRT clinical ESCC specimen would use to analyze E- and N-cadherin levels. However, due to the restriction of clinical specimen collection procedure in our faculty, specimens acquisition as mentioned were not easy. Alternatively, we examined N-cadherin expressions by immunohistochemical staining using endoscopic biopsy samples, which were prior to receive CCRT treatment, to investigate the association of N-cadherin expressions with CCRT response. Total 137 tissue sections were analyzed, each slide was assigned a score by the staining intensity: 0 (no staining, N = 49), 1 (weak staining, N = 31), 2 (moderate staining, N = 21), and 3 (strong staining, N = 34), representative photos were shown in Figure 5A. A total of 86 tissue specimens were found to exhibit high N-cadherin expression, 49 tissue specimens were without N-cadherin staining, and 2 tissue specimens were broke (Figure 5B). Among these 86 samples with higher N-cadherin expression showed a high percentage, approximately 64%, for a poor response to CCRT treatment. Together with the results of in vitro study using ESCC cells in Figure 2, Figure 3 and Figure 4, it suggested N-cadherin levels may determine the response to CCRT treatment.

## 3. Discussion

The biological role of miR-200b and miR-200c contributes to EMT have been studied [32]; however, its potential pathological roles in developing disease, particular in ESCC patients, and the association of their clinical relevance with CCRT treatment are still under investigation. Our current study provided some novel insights in the pathological roles of miR-200b and miR-200c contributes to EMT in ESCC. Figure 5C illustrates the potential mechanism of ESCC patients acquire CCRT resistance is through miR-200 family mediated epithelial–mesenchymal characteristic. During the interval of CCRT therapy, CCRT resistance ESCC cells acquired mesenchymal characteristic by the loss of miR-200 family members expression such as miR-200b and miR-200c. Down-regulated miR-200b and miR-200c can trigger the conversion of epithelial to mesenchymal type via the snail and slug expressions. Re-introducing miR-200b and miR-200c into CCRT resistance cells not only reverse cells morphology from mesenchymal to epithelial type, but also sensitize refractory cells to CCRT. Conversely, suppressed miR-200b and miR-200c can cause N-cadherin to be expressed, promote control ESCC cells lose the epithelial characteristic, and then confer cells harbor the resistance property to CCRT. Besides, we examined the expression levels of mesenchymal marker (N-cadherin) in clinical specimens, and explored it could be a potential indicator to predict the therapeutic outcome to CCRT treatment in ESCC patients.

Our established CCRT resistance model evidence ESCC cells respond to CCRT treatment stress and evolutionarily develop to CCRT resistance by enabling cells re-adopt mesenchymal feature. miRNA-200 family expression is downregulated in this pathological alternation of epithelial–mesenchymal characteristic (Figure 1 and Figure 2). By manipulating miRNA-200 family members expressions, either in CCRT resistance or in parental ESCC cells (Figure 3 and Figure 4) clearly established the cause-and-effect of miRNA-200 family members in the development of CCRT resistance. miRNA-200 family members alter epithelial–mesenchymal characteristic and then modulate cell sensitivity to CCRT, instead of CCRT resistance leads to the consequence of miRNA-200 loss.

Esophageal cancer is a fatal upper gastrointestinal malignancy in the world and requires multimodal therapy. There are around 18 to 40% ESCC patients were diagnosed with distant organ metastasis such as lungs, liver, bone, or nonregional lymph nodes [33]. The treatment options for esophageal cancer include endoscopic resection, surgery, and chemoradiotherapy [34]. Recently, pre- and post-operative chemotherapy, as well as concurrent chemoradiation therapy (CCRT), have greatly prolong the survival time of patients with locally advanced esophageal tumors. For ESCC patients with local invasion and metastasis, which are not proper for surgery, CCRT is an effective first-line therapeutic regimen [6,35,36,37], CCRT therapeutic outcome is much better than chemotherapy alone for patients with stage IV ESCC [38]. However, the incidence of acquired CCRT resistance leads to the failure of tumor treatment and tumor recurrence is increasing over year [39,40,41]. Mechanistically, PI3K/Akt/mTOR signaling pathway has been shown to regulate cell growth, differentiation, proliferation, metastasis and chemoradiotherapy sensitivity in ESCC [42]. Both downregulation of BMI-1, or IDH2 lead to the suppression of PI3K/Akt/mTOR activity, which can increase radio-sensitivity [43,44]. In addition, ERBB3 and SIX1 can, respectively, contribute to chemoradiotherapy resistance through activation of the Akt signaling pathway [45,46], suggesting the important role of Akt in therapeutic efficiency. Furthermore, defects in DNA repair and damage response genes such as RAD51, KU80 SIRT1, NFAT5, and REV3L [47], or ESCC tumor cells expressed higher CLDN4 to harbor stem-like properties [48] are also the potential CCRT resistance mechanisms.

The process of EMT mechanism is reversible. Cells can change their phenotypes by responding to microenvironmental alterations and aberrant stimulation such as hypoxia, inflammation, and increased tissue stiffness [49]. In addition to miRNA, EMT can be regulated by numerous inside molecular signals including lncRNA, NF-ĸB, Wnt, and PI3K/Akt signaling pathway [50,51]. Hypoxia-induced lncRNA RP11-390F4.3 [52], Wnt/β-catenin signaling [53], and IKK-2/IκBα/NF-κB pathway [54] in breast cancer can regulate multiple EMT regulators such as Snail, Twist1, and ZEB1/2 to promote tumor metastasis. Drug resistance and tumors with distal metastasis are the two major obstacles to the success of cancer treatment. The association of drug resistance and cancer invasion/metastasis was uncovered [55,56]. EMT process can significantly contribute to chemoresistance such as cisplatin, doxorubicin, and EGFR-TKI treatment in many cancer types, including lung, breast hepatocellular carcinoma, ovarian, and oral cancers [57,58,59]. Including but not limited to our current study, the pathological role of miRNA-200 family regulates tumorigenesis, angiogenesis, chemo-sensitivity, and involves in the process of EMT to promote tumor metastasis have been confirmed [60]. Taken these together and based on our findings, it is possible that miRNA-200 family might be a double-edged sword in ESCC tumors; in addition to confer cells resist to CCRT treatment, but also to enhance these refractory cells metastasis. In our ESCC model, it has to furtherly investigate whether CCRT resistance cells exhibit an enhanced metastasis capacity; however, this possibility could be supported by a clinical study that ESCC patients fail to CCRT treatment have higher incidence of tumor recurrence and metastasis [61].

Recently, the application of targeting miRNA as a therapeutic approach is under phase I/II clinical trial, many drugs are developed to target disease-related miRNAs such as miR-34 in liver cancer, lymphoma, melanoma; miR-16 in lung cancer, and miR-92 in heart failure [62]. In the present study, our results demonstrated ectopically increase miR-200 family expressions enable resistance cells to restore the sensitivity to CCRT treatment, providing a further support for targeting miR-200 family as future therapeutics in ESCC. In addition, some miRNAs in Figure 2E such as miR-205-5p and miR-16-5p have been shown to associate with gemcitabine sensitivity, proliferation and invasion in breast cancer [63,64]; miR-323a-5p plays a tumor-suppressive role in neuroblastoma [65], whereas miR-92b-3p acts as a tumor suppressor in pancreatic cancer [66]. The precise roles of these downregulated miRNAs in ESCC or in the development of acquired CCRT resistance will have to be further addressed in future.

In conclusion, the present results established a potential mechanism of CCRT resistance refers to the alteration of epithelial–mesenchymal feature in ESCC by microRNA. Through modulating the status of epithelial–mesenchymal type in ESCC cells can affect CCRT sensitivity. Moreover, according to the results of clinical specimen analysis, the EMT status in tumor cells might a potential indicator to predict the responsive outcome of CCRT treatment.

## 4. Materials and Methods

### 4.1. Cell Culture and Reagents

CE48T, KYSE70, KYSE170, and KYSE510 cell lines were obtained from professor Yi-Ching Wang (National Cheng Kung University, Tainan, Taiwan). All ESCC cell lines were cultured in RPMI-1640 medium containing 10% fetal bovine serum (FBS; Hyclone, Logan, YT, USA) and 100 units/mL penicillin/streptomycin (Caisson, North Logan, UT, USA). All cells were maintained at 37 °C in an atmosphere containing 5% CO_2_. The artificial miRNA precursors were purchased from Applied Biosystems (Applied Biosystems, Branchburg, NJ, USA): hsa-miR-200b-3p-MIMAT0000318 (UAAUACUGCCUGGU AAUGAUGA); hsa-miR-200c-3p-MIMAT0000617 (UAAUACUGCCGGGUAAUG AUGGA). Inhibitor molecules were purchased from Exiqon (Exiqon A/S, Vedbaek, Denmark).

### 4.2. CCRT Resistant Cell Lines Establishment

Parental CE48T and KYSE70 ESCC cell lines were used to generate CCRT resistant cell line according to previous study [47]. Briefly, ESCC cells were established through a stepwise increase in cisplatin and irradiation. Cells were exposed to radiation dose of 5 Gy, the dosage of cisplatin was gradually increased from 0.1 μM until reaching a concentration of 20 μM. After treatment, cells were allowed to recover, and then the next exposure was given when cells reached at least 50–60% confluency. A total of 75 Gy was given in 15 fractions of 5 Gy each. Cell lysates were collected for Western blotting analysis at 5th, 10th, and 15th CCRT treatment.

### 4.3. Western Blotting Analysis

For Western blotting assay, protein lysates were loaded onto a 10% SDS-polyacrylamide gel for electrophoresis and transferred to a PVDF membrane. Proteins were identified by incubating the membrane with primary antibodies followed by horseradish peroxidase-conjugated secondary antibodies and enhanced chemiluminescence solution (NEN Life Science, Boston, MA, USA). The primary antibodies used in this study were E-cadherin (Cell Signaling, Danvers, MA, USA), N-cadherin (GeneTex, Irvine, CA, USA), snail (Abnova, Taipei, Taiwan), slug (Abnova), and β-actin (GeneTex).

### 4.4. Immunofluorescence Assay

For E-cadherin staining, parental and CCRT resistant cells were seeded into cover slides in a 12-wells plate. Afterwards, cells were fixed by 4% paraformaldehyde at room temperature for 20 min, and then washed the cover slides 3 times with 1X PBS to remove paraformaldehyde solution. The 0.5% Triton X-100 in 1X PBS was added into cells for permeabilization process. After 30 min incubation, cells were washed by 1X PBS 3 times and then blocked by 10% FBS at room temperature for 60 min. The 1:300 diluted E-Cadherin primary antibody was incubated with cells overnight at 4 °C. The next day, removed primary antibody solution, washed cells and then incubated cells with fluorescent dye-conjugated secondary antibody (Goat anti-rabbit IgG Alexa 488, ab150077, 1:800) for 60 min. Hoechest solution (#33342, Sigma-Aldrich) was used to stain cell nuclei. Finally, cells were mounted and analyzed by microscope.

### 4.5. Colony Formation Assay

The colony formation assays were performed on a six-well plate. The parental and CCRT-resistant cells were seeded, and the plates were incubated at 37 °C in 5% CO_2_. After 12–14 days, the plates were stained with 1% crystal violet. A single colony was defined to consist of at least 30 cells. The colony number in each well was counted after staining.

### 4.6. MTT Assay

The 1 × 10^4^ ESCC cells were plated onto 96-well plates and cultured overnight for complete cell attachment. The medium was removed and replaced with 0.33 mg/mL 3-(4,5-dimethylthiazol-2-yl)-2,5-diphenyl-5H-tetrazolium bromide (MTT, Sigma, St Louis, MO, USA) in RPMI for 2 h. After incubation, the absorbance at 570 nm was determined using an ELISA reader. To verify the effect of the CCRT response, cells were treated with different dosages of cisplatin combined with 5 Gy irradiation.

### 4.7. MicroRNA Array and Analysis

Total RNAs were isolated from parental and CCRT resistant cells by TRIzol reagent (Invitrogen, Waltham, MA, USA). The expression of microRNAs and mRNAs in these cells were evaluated by Human OneArray system (HOA 6.1). All data were normalized to control miRNA. The quantification data are shown as the ratio of CCRT resistant to parental fluorescence intensity for each miRNA.

### 4.8. Quantitative Real-Time PCR (Q-PCR)

Total RNA isolation and reverse transcription were conducted from cells. The amplification and detection of specific products were performed with the cycle profile according to the Fast SYBR Green Master Mix and a StepOne real-time PCR system. The target PCR Ct values were normalized to the internal control U6 Ct values.

### 4.9. Clinical Specimens

Primary esophageal tumor tissues were obtained from the National Cheng Kung University Hospital (Tainan, Taiwan). One hundred and thirty-seven patients donated tissues before CCRT treatment to evaluate the expression of N-cadherin. This study received Institutional Review Board approval (IRB numbers: BR-100-087). The carcinoma samples were obtained from a resection of the esophageal tumors, which were histologically examined for the presence of tumor tissue in hematoxylin and eosin-stained sections.

### 4.10. Immunohistochemistry Analysis

The clinical specimen was dewaxed and rehydrated. Antigen retrieval was performed by heating the sections in 0.01M sodium citrate buffer (pH 6.4). Endogenous peroxidase activity was blocked by immersion in 3% H2O2/methanol. Then, the sections were blocked in 5% normal goat serum/1x PBS. The sections were incubated with an E-CADHERIN (Cell Signaling) or N-CADHERIN (GeneTex) antibodies followed by a biotinylated secondary antibody. The signals were revealed using the standard avidin-biotin-peroxidase complex method (ABC Elite) according to the manufacturer’s instructions. Immunoreaction products were visualized using 3,3′-diaminobenzidine substrates (DAB, Sigma).

### 4.11. Statistical Analysis

All observations were confirmed by at least three independent experiments. We used a two-tailed, paired Student’s *t*-test for all pair-wise comparisons. Data were analyzed using GraphPad Prism 6 software (GraphPad Software, San Diego, CA, USA).

## Data Availability

Data and materials used for this paper are available upon reasonable request.
